# Supplementary material for: Treatment Resistant Persister Cells Exploit Macrophage Lipid Metabolism to Sustain Glioblastoma Growth
Source: bioRxiv. 2025 Jun 10:2025.06.07.658345. Preprint. [Version 1] doi: 10.1101/2025.06.07.658345 (PMC12259058; doi:10.1101/2025.06.07.658345)

Supplemental Figure 1

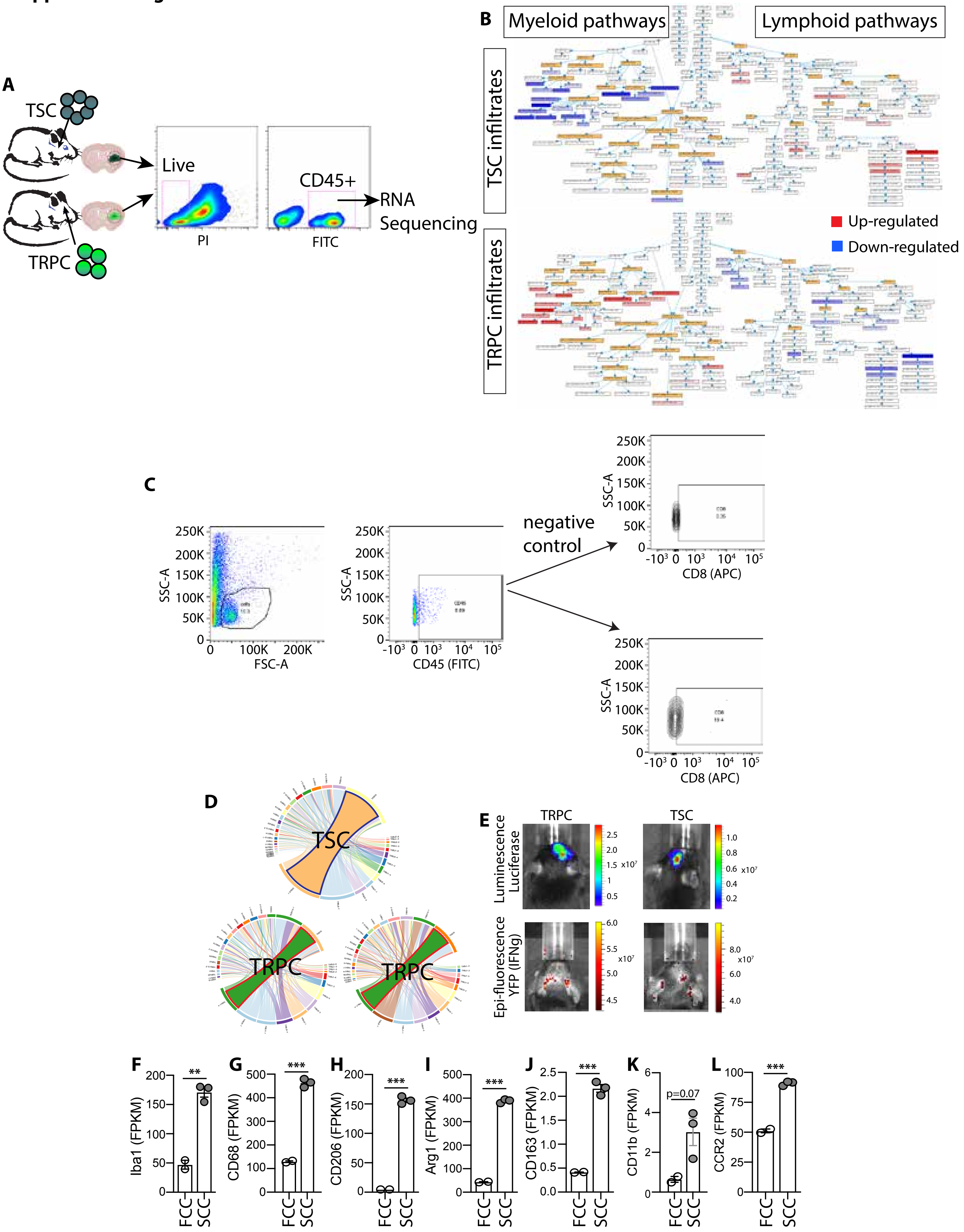

Supplemental Figure 2

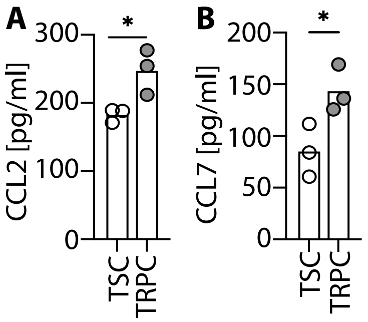

Supplemental Figure 3

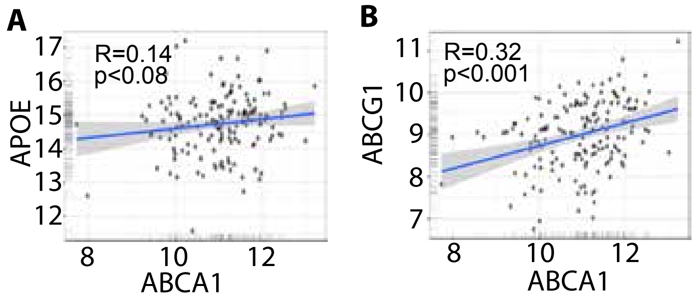

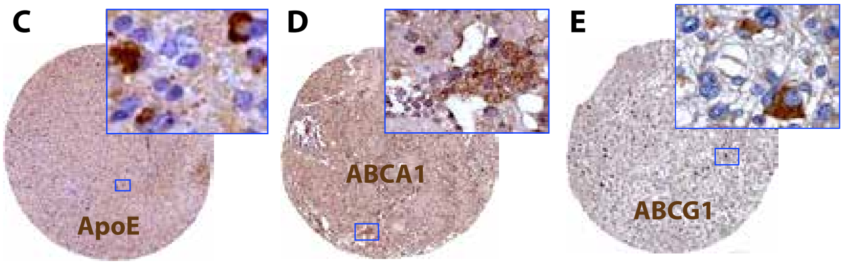

Glioma (HPA065539)
Patient id: 2790
https://www.proteinatlas.org/ENSG00000130203-APOE/pathology/glioma#img

Glioma (CAB069889)
Patient id: 2962
https://www.proteinatlas.org/ENSG00000165029-ABCA1/pathology/glioma#img

Glioma (HPA031471)
Patient id: 3203
https://www.proteinatlas.org/ENSG00000160179-ABCG1/pathology/glioma#img

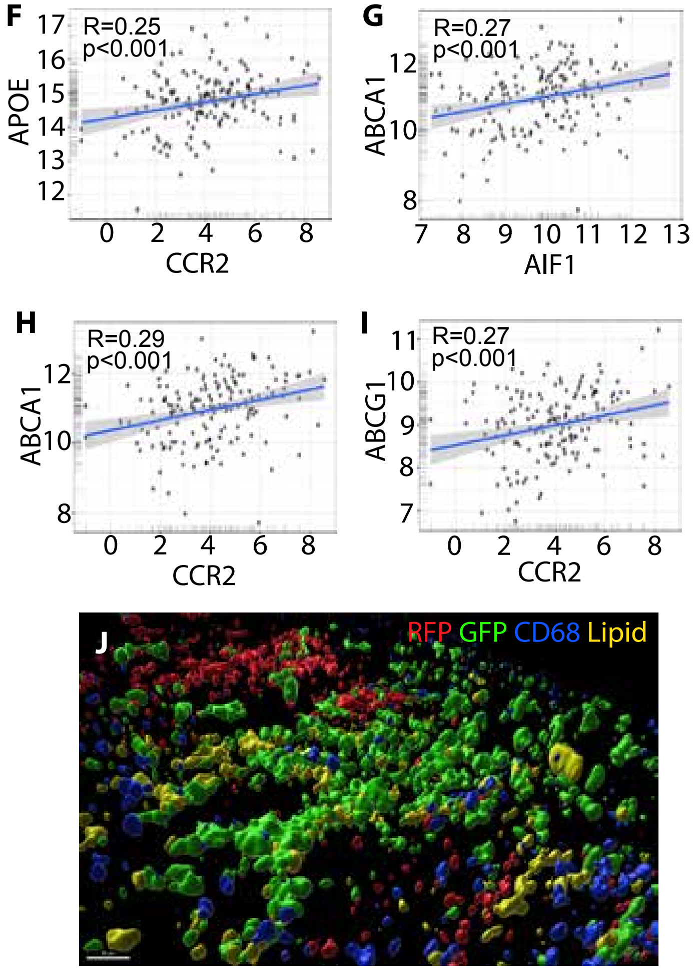

Supplemental Figure 4

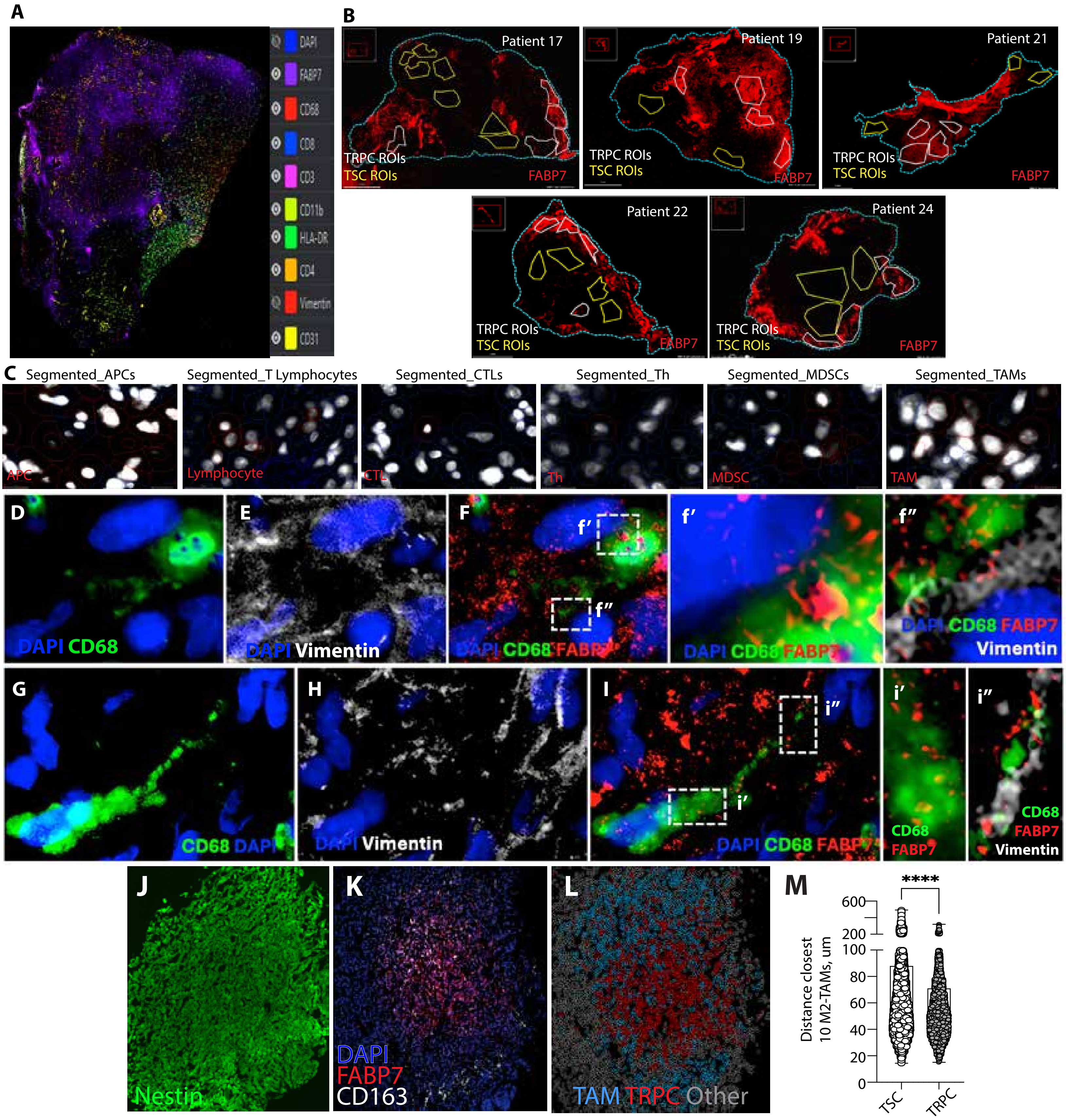

Supplemental Figure 5

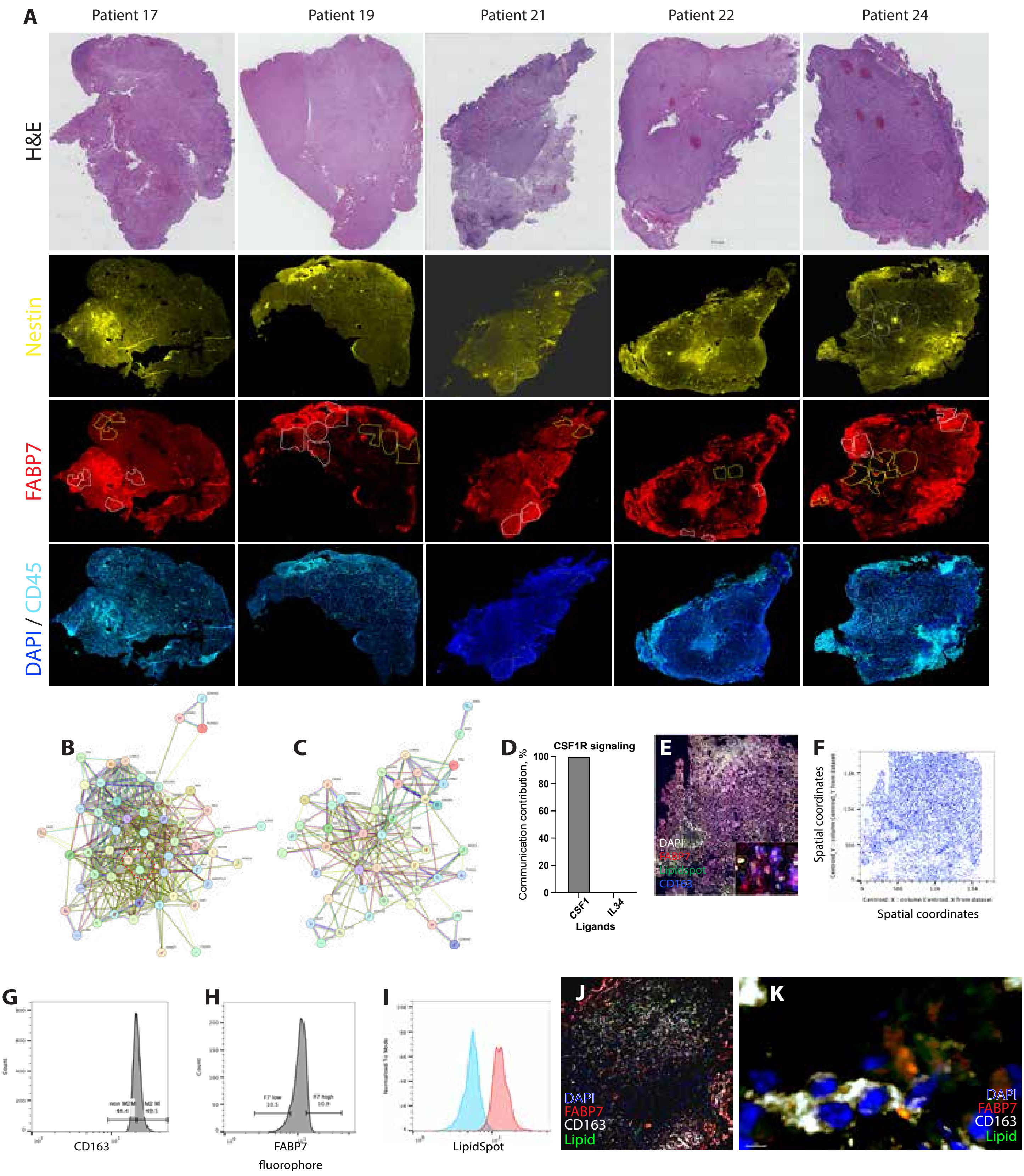

Supplemental Figure 6

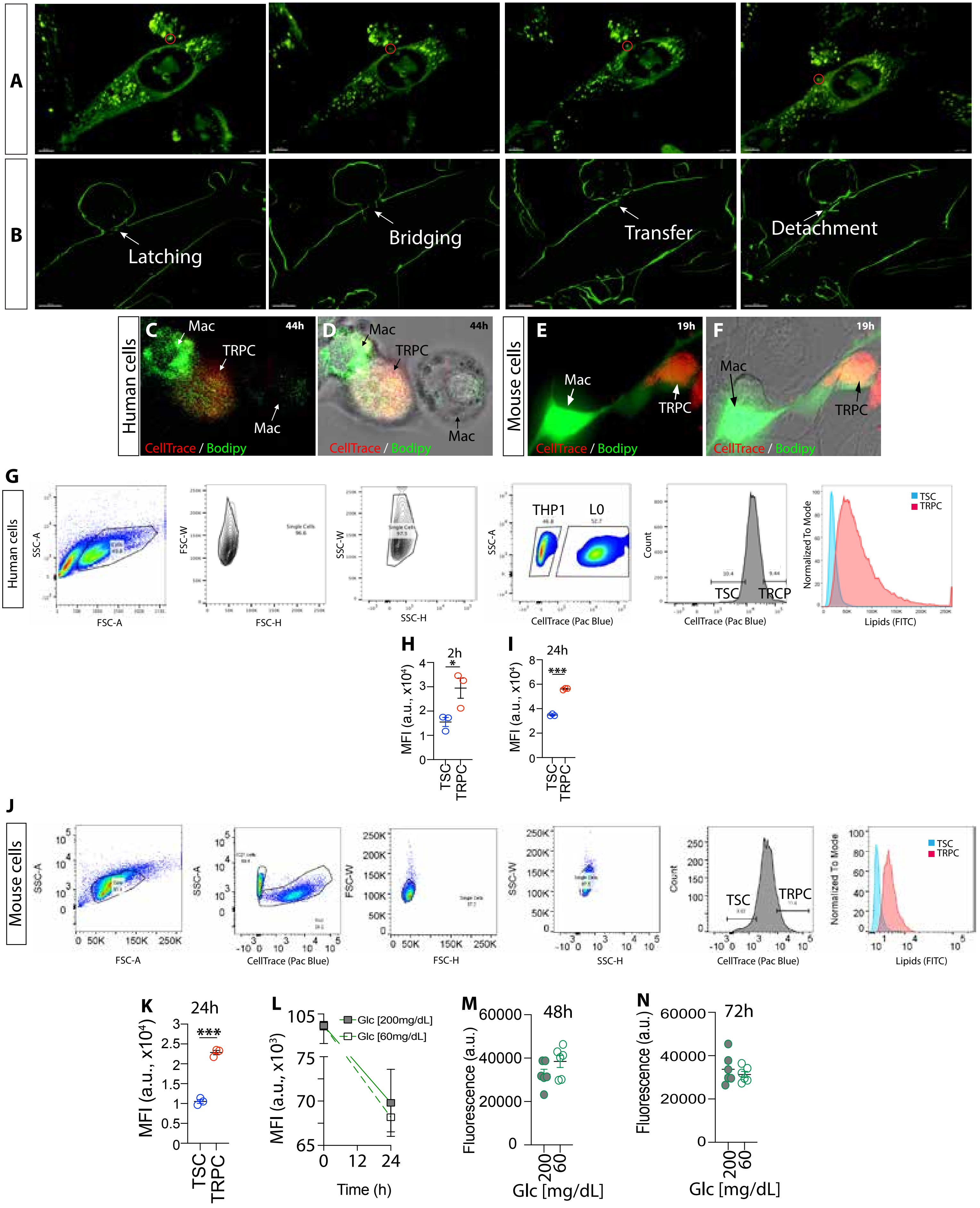

Supplemental Figure 7

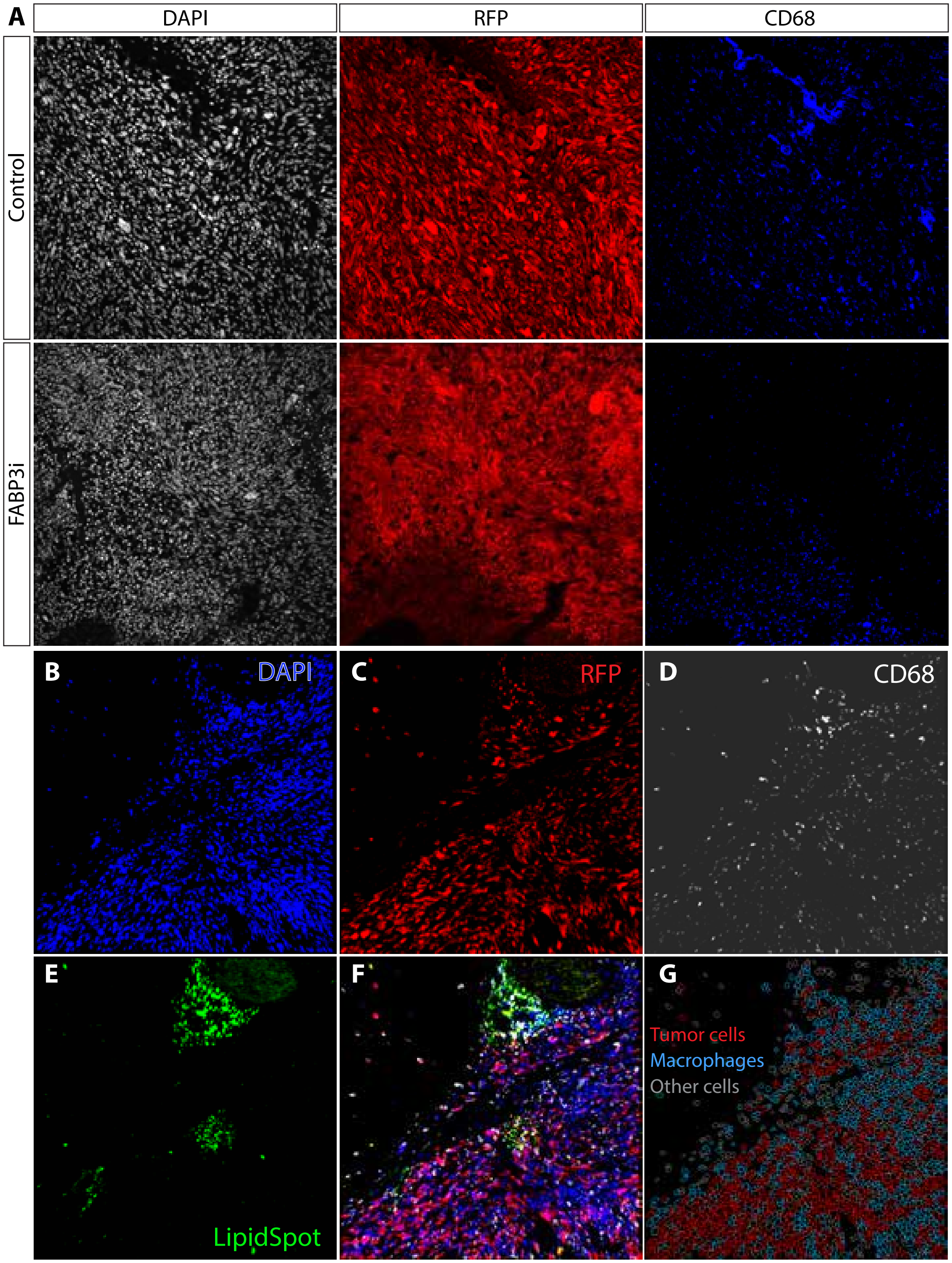

Supplemental Figure 8

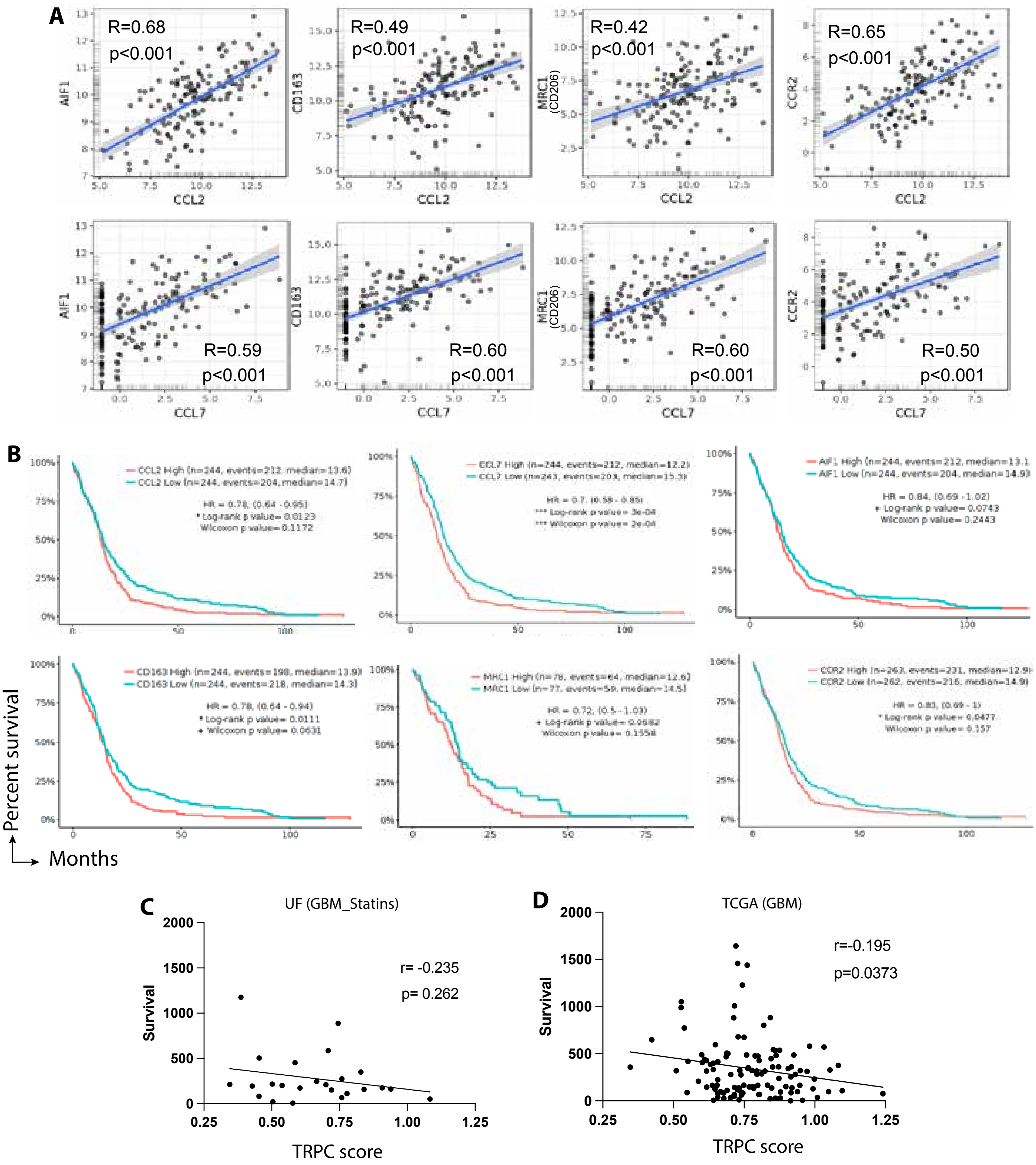

Supplement: Supplement 1 — Supplemental Figure 1. A) Graphical representation illustrating intracranial injection of TRPC and TCS KLuc cells into mice and generated tumors. The flow cytometry gating algorithm is then used to sort live immune cell population for downstream RNA sequencing analysis. (TSC n=2, TRPC n=3) B) Immune pathway enrichment analysis of ImmInfs within mTRPC/mTSC tumors C) Gating strategy of flow-based analysis determining infiltrating immune cells and proportion of CD8 cells within the population with negative control gating. D) Circos plot from bulk TCR sequencing analysis measuring the amplification and unique arrangements of the V and J segments of the TCRβ chain showing higher frequency of specific dominant clonotype within the TSC tumor population. E) Tumor burden in BL6 mice implanted with TRPCs or TSCs were quantified three weeks post injection by bioluminescence based on luciferase activity. In vivo fluorescent imaging of the YFP channel in both groups was acquired to serve as background signal for the imaging performed with GREAT mice. F-L) FPKM values from paired-end RNA-seq analysis were used to compare (F) Iba1 (p<0.01), (G) CD68 (p<0.0001), (H) CD206, (I) Arg1, (J) CD163, (K) CD11b, (L) CCR2 expressions in TRPC and TSC tumors (Unpaired t-test) Supplemental Figure 2. A-B) Serum level expressions of CCL2 and CCL7 using ELISA. (n=3, 3; Unpaired t-test) Supplemental Figure 3. A-B) Correlation analysis using bulk RNA sequencing data from GBM TCGA, with Pearson’s correlation between (A) APOE and ABCA1, and (B) ABCA1 and ABCG1 C-E) IHC from the protein atlas of GBM patients showing tissue expression of (C) ApoE, (D) ABCA1, and (E) ABGC1 F-I) Correlation analysis using Pearson’s correlation coefficient between (J) ApoE and CCR2, G) ABCA1 and AIF1, H) ABCA1 and CCR2, and I) ABCG1 and CCR2 (GBM TCGA) J) 3D rendering of the GBM TME showing the lipid deposition and TAM accumulation within the TRPC region. (n=5) Supplemental Figure 4. A) Representative image of a human GBM [file media-1.pdf]
